# Cross-sectional analysis of clinical aspects in patients with long-COVID and post-COVID syndrome

**DOI:** 10.3389/fneur.2022.979152

**Published:** 2022-10-14

**Authors:** Hannah Schulze, Jeyanthan Charles James, Nadine Trampe, Daniel Richter, Thivya Pakeerathan, Nadine Siems, Ilya Ayzenberg, Ralf Gold, Simon Faissner

**Affiliations:** Department of Neurology, St. Josef-Hospital, Ruhr-University Bochum, Bochum, Germany

**Keywords:** COVID-19, post-COVID syndrome, fatigue, smell disorder, depression, anxiety, SARS-CoV-2

## Abstract

**Objective:**

Regarding pathogenesis, clinical manifestations, at-risk individuals, and diagnostic methods for stratifying patients for therapeutic approaches, our understanding of post-COVID syndrome is limited. Here, we set out to assess sociodemographic and clinical aspects in patients with the long-COVID and post-COVID syndrome.

**Methods:**

We performed a cross-sectional analysis of patients presenting at our specialized university hospital outpatient clinic. We assessed patients' clinical presentation, fatigue, symptoms of depression and anxiety, and impairment of smell.

**Results:**

A total of 101 patients were included (73.3% female), of whom 78.2% had a mild course of COVID-19. At presentation, 93.1% suffered from fatigue, 82.2% from impaired concentration, and 79.2% from impaired memory, 53.5% had impaired sleep. The most common secondary diagnosis found in our cohort was thyroid disease. Fatigue analysis showed that 81.3% of female and 58.8% of male patients had severe combined fatigue. Female gender was an independent risk factor for severe fatigue (severe cognitive fatigue OR = 8.045, *p* = 0.010; severe motor fatigue OR = 7.698, *p* = 0.013). Males suffered from more depressive symptoms, which correlated positively with the duration of symptom onset. 70.3% of patients with anamnestic smell impairment had hyposmia, and 18.9% were anosmic.

**Interpretation:**

Most long-COVID patients suffered from severe fatigue, with the female sex as an independent risk factor. Fatigue was not associated with symptoms of depression or anxiety. Patients with long-COVID symptoms should receive an interdisciplinary diagnostic and therapeutic approach depending on the clinical presentation.

## Introduction

The Severe Acute Respiratory Syndrome Coronavirus 2 (SARS-CoV-2) has led to a global pandemic. A “standard course” with specific disease phases has not yet been identified. The SARS-CoV-2 infection can be divided into three phases (1): During the acute phase, active viral replication and the initial host response occur. This phase can be accompanied by clinical manifestation but can also be asymptomatic or clinically inapparent in 67% (2). The acute phase can last days to weeks (3). Two to five weeks after the host's acute infection and virus elimination, hyperinflammation may occur in some patients, even in organ systems unaffected by the virus. This disease entity is subsumed as a multisystemic inflammatory syndrome and may present with gastrointestinal, cardiovascular, dermatologic, pulmonological, and neurologic symptoms (4). Up to 87.4% of patients having recovered from Coronavirus Disease 2019 (COVID-19) experience symptoms that persist for a protracted period from the fourth week after primary infection (1, 5), summarized as long-COVID (after the fourth week) or post-COVID syndrome (after the twelfth week). There is growing evidence that a substantial number of patients still suffer from persisting symptoms months after the acute phase of the disease. The spectrum of symptoms of long-COVID and the post-COVID syndrome is wide, ranging from fatigue, depression, and neuropsychological deficits such as memory or word-finding disorders to myopathies, muscle weakness, or sleep disturbances, amongst others (5–7). Until now, it remains incompletely understood which factors might predispose to the development of the post-COVID syndrome and whether it indeed is a new unique entity.

Mechanisms of the post-COVID syndrome are still under investigation. Causal factors are thought to include an immune system imbalance that persists long after the disease, leading to the release of pro-inflammatory cytokines such as Tumor Necrosis Factor α (TNF-α), Interferon-1β (IFN-1β), and nitrogen metabolites such as inducible Nitric Oxide Synthase (iNOS), driving the expression of pro-inflammatory microglia in the central nervous system, among others (8). Pathophysiological considerations also include the formation of a subset of exhausted T cells and dedifferentiated monocytes observed in patients with neurological manifestations of COVID-19 (9) or anti-idiotypic antibodies (10).

Initial cross-sectional studies of patients who have experienced COVID-19 disease and have persistent symptoms show that patients who develop post-COVID syndrome usually have a mild course of the disease and suffer primarily from mood swings, fatigue, and perceived cognitive impairment (11). Until now, it remains elusive whether there might be sociodemographic characteristics or certain comorbidities driving the risk of developing the post-COVID syndrome.

In this cross-sectional non-interventional study of patients with the long-COVID and post-COVID syndrome, we characterized patients with COVID-19 disease who presented to our specialized university hospital neurological outpatient clinic regarding sociodemographic variables and clinical phenotype with a focus on fatigue, symptoms of depression, anxiety, and impairment of smell. First, we wanted to characterize patients with long-COVID regarding sociodemographic variables, secondary diseases, symptoms following COVID-19, and their duration. Second, we performed a psychometric quantification of fatigue and depressive symptoms in patients with long-COVID and post-COVID syndromes to assess whether fatigue and affective symptoms might be associated with the severity of COVID, age, sex, and prior psychiatric disorders. Those data may help to better understand neurological manifestations of long-COVID syndrome and to guide directions for therapeutic approaches.

## Methods

Patients were included after having written informed consent to participate. The study was conducted in accordance with the Helsinki Declaration of 1975 and was approved by the local ethics committee of Ruhr-University Bochum (20–6827; 21–7423). Patients have been recruited at our specialized neurology long-COVID clinic at the Ruhr-University Bochum, St. Josef Hospital. Patients were recruited from January 2021 onwards. All patients were examined by an experienced, board-certified senior neurology consultant. Patients presented after a referral from a resident specialist or general practitioner. Only patients with long-COVID or post-COVID syndrome were recruited for the study. We included *n* = 101 patients older than 18 years. The COVID-19 infection was at least 2 months before the presentation. The majority of patients presented more than 3 months after infection (87/98 patients, 88.8%). To understand the long-term effects of a mild or moderate disease, only patients with a moderate disease corresponding to less than six points in the World Health Organization (WHO) clinical progression scale were included (12). Demographic data, disease duration, symptoms, family history, medication history, and previous therapies were recorded.

### Assessment of cognitive and motor fatigue

We performed the Fatigue Scale for Motor Function and Cognition (FSMC) to objectively measure fatigue symptoms. The FSMC is a self-report measure developed to characterize motor and cognitive fatigue symptoms into mild, moderate, and severe fatigue in patients with Multiple Sclerosis. It is also available in German (13). Patients who did not fill out all the items of the FSMC were excluded from the analysis. Accordingly, 36 patients were excluded from the evaluation, and the questionnaires of 65 patients were analyzed.

### Hospital anxiety and depression scale

Psychometrics of affective symptoms were performed using the German version of the self-reported *Hospital Anxiety and Depression Scale* (HADS-D), consisting of a questionnaire with 14 items (14). Depression and anxiety symptoms are measured by seven items each, and the two subscales are interpreted independently. Twelve incomplete questionnaires had to be removed (*n* = 89 analyzed).

### Odor testing

The odor testing was performed using Sniffin' Sticks (15, 16). An experienced examiner performed the test. The correct identification of 13 odors or more was considered normosmia, 8 to 12 odors indicated hyposmia, and seven or fewer were considered anosmia (17).

### Statistical analysis

Data were presented as shown in the respective figure legends. We compared demographics, clinical characteristics, and outcomes between patients with univariate analysis using appropriate nonparametric tests (Mann-Whitney–*U* test, Chi-squared test). Furthermore, we applied two multivariable logistic regression models to calculate odds ratios (OR) and the corresponding 95% confidence intervals (CI) for each outcome of severe motor or cognitive fatigue. A value of ≥32 points in the motor subscale of FSMC and ≥34 points in the cognitive subscale of FSMC was used as a cut-off to indicate severe motor or cognitive fatigue, respectively. Statistical analysis was performed with GraphPad Prism (version 9.2.0) and SPSS 27.0 for Mac. *P* < 0.05 was defined as the level of statistical significance.

## Results

### Sociodemographic characteristics and comorbidities

We included 101 patients, and the proportion of female patients was 73.3% (*n* = 74). The mean age of the total cohort was 50.2 years (range 19–84; Supplementary Figure 1). The time from the onset of COVID symptoms to presentation to our outpatient clinic averaged 220.1 days (SD: 118.26, Range: 60–554, *n* = 98). At the presentation time, 83.0% of patients lived in a partnership. Only 2.7% of patients were in education or had no vocational degree at the presentation time. 58.8% were on sick leave and unable to work (Table 1).

**Table 1:** Sociodemographic data.

	**Total**	**Male**	**Female**
Number of patients	101	27 (26.7%)	74 (73.3%)
**Age (** ***years*** **)**			
Median	51	54	50.5
Mean	50.2	52.7	49.3
SD	12.9	14.2	12.4
Range	19–84	19–77	24–84
**The time between symptom onset and presentation to the outpatient clinic (** ***days*** **)**	*n =* 98	*n =* 25	*n =* 73
Median	201	214	184
Mean	220.1	225.4	218.3
SD	118.3	101.4	124.1
Range	60–554	72–492	60–554
**Marital status**			
Cohabitation/permanent partnership/married	73/88 (83.0%)	22/25 (88.0%)	51/63 (81.0%)
Single/divorced/widowed	15/88 (17.0%)	3/25 (12.0%)	12/63 (19.0%)
**Highest degree**			
Completion of compulsory basic secondary schooling	5/46 (10.9%)	2/13 (15.4%)	3/33 (9.1%)
General certificate of secondary education	13/46 (28.3%)	3/13 (23.1%)	10/33 (30.3%)
Technical college entrance qualification	9/46 (19.6%)	1/13 (7.7%)	8/33 (24.2%)
General qualification for university entrance	19/46 (41.3%)	7/13 (53.8%)	12/33 (36.4%)
**Highest professional qualification**			
No training completed/still in training	2/75 (2.7%)	1/19 (5.3%)	1/56 (1.8%)
Completed vocational training	51/75 (68.0%)	9/19 (47.4%)	42/56 (75.0%)
Completed university education	22/75 (29.3%)	9/19 (47.4%)	13/56 (23.2%)
**Professional** ***situ*****ation prior to** ***COVID*****-19 infection**			
Training/further education/retraining	1/82 (1.2%)	–	1/61 (1.6%)
Employment (full-time)	56/82 (68.3%)	16/21 (76.2%)	40/61 (65.6%)
Employment (part-time)	11/82 (13.4%)	–	11/61 (18.0%)
Early retirement	2/82 (2.4%)	1/21 (4.8%)	1/61 (1.6%)
Jobseeker	–	–	–
Housewife/houseman	2/82 (2.4%)	–	2/61 (3.3%)
Retirement	9/82 (11.0%)	4/21 (19.0%)	5/61 (8.2%)
Incapacitated	1/82 (1.2%)	–	1/61 (1.6%)
**The professional** ***situ*****ation at the time of the presentation**			
Training/further education/retraining	1/52 (1.9%)	–	1/37 (2.7%)
Employment (full-time)	21/52 (40.4%)	8/15 (53.3%)	13/37 (35.1%)
Employment (part-time)	11/52 (21.2%)	–	11/37 (29.7%)
Early retirement	1/52 (1.9%)	–	1/37 (2.7%)
Jobseeker	3/52 (5.8%)	1/15 (6.7%)	2/37 (5.4%)
Housewife/houseman	3/52 (5.8%)	–	3/37 (8.1%)
Retirement	10/52 (19.2%)	5/15 (33.3%)	5/37 (13.5%)
Incapacitated	2/52 (3.8%)	1/15 (6.7%)	1/37 (2.7%)
**Sick leave at the time of presentation**	20/34 (58.8%)	5/10 (50.0%)	15/24 (62.5%)

**Figure 1:**
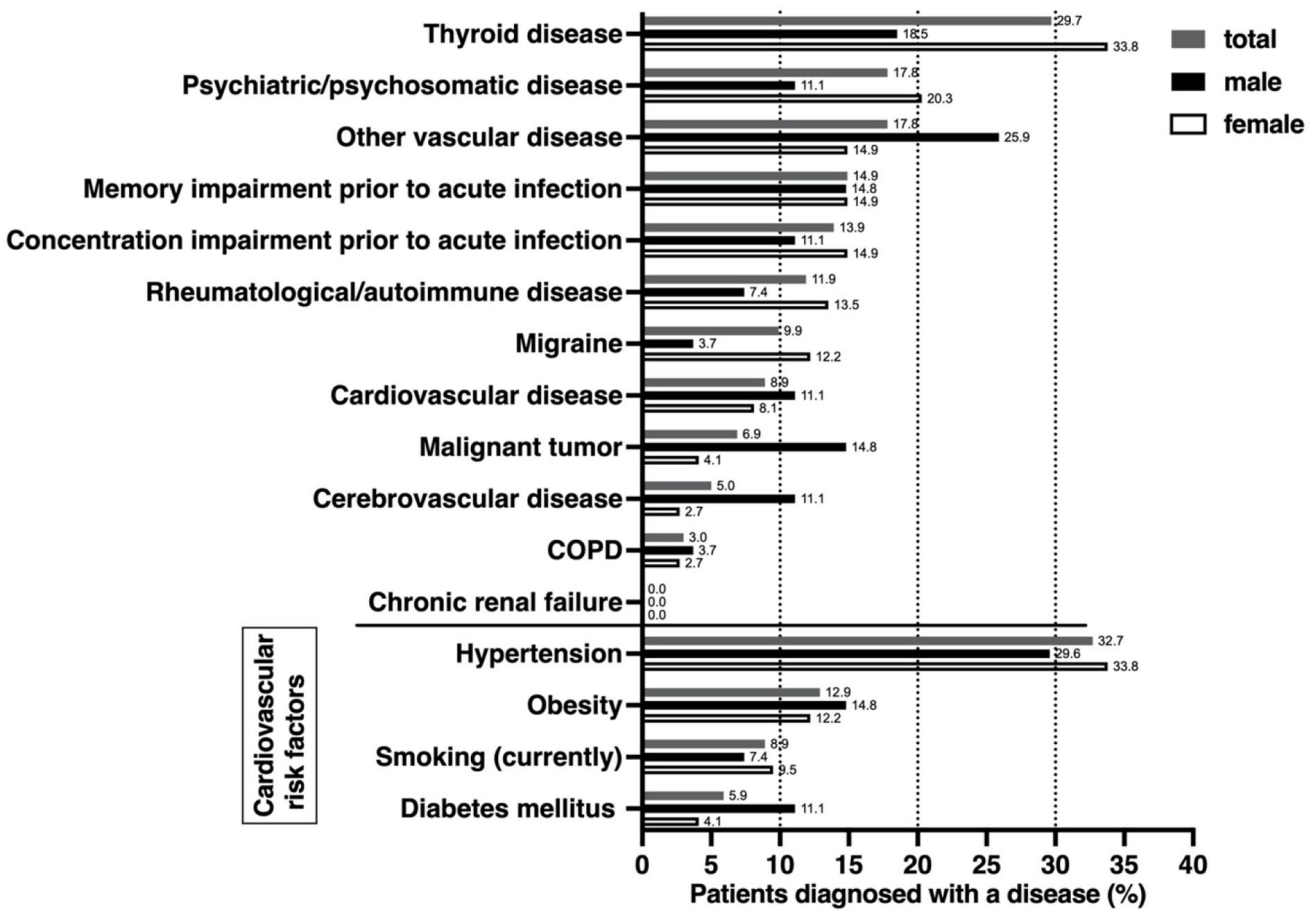
Comorbidities, self-reported cognitive impairment, and cardiovascular risk factors prior to acute COVID-19 infection. Data were derived from a self-questionnaire, covering previous comorbidities from various organ systems as well as the most important cardiovascular risk factors. Impairment of cognition or concentration was self-assessed by patients. Differences between females and males were analyzed using a nonparametric two-tailed Mann-Whitney test, which showed no differences.

The most common secondary diagnosis was thyroid disease (29.7%), with a proportion of 33.8% among females compared to a proportion of 18.5% in males (Figure 1). Thyroid disorders were followed by psychiatric or psychosomatic secondary diagnoses with a total proportion of 17.8% (females 20.3%, males 11.1%). Among the psychiatric or psychological history of patients included, depression was the most common pre-existing condition (16/101, 15.8%), followed by adjustment disorder (2/101, 2,0%), condition after borderline personality disorder (1/101, 1.0%), condition after narcotic abuse (1/101, 1,0%), anxiety disorder (1/101, 1,0%), post-traumatic stress disorder (1/101, 1,0%) and panic disorder (1/101, 1.0%). Some patients had more than one diagnosis. 14.9% of patients reported memory impairment, and 13.9% reported concentration impairment before the onset of COVID infection. Rheumatologic/autoimmune secondary diagnoses were reported by 11.9%. Pre-existing rheumatologic or autoimmunologic conditions included psoriasis (2/101; 1.0%), Bechterew's disease (1/101, 1.0%), Hashimoto's thyroiditis (4/101, 4.0%), psoriatic arthritis (1/101, 1.0%), rheumatoid arthritis (1/101, 1.0%), multiple sclerosis (2/101, 2.0%), fibromyalgia syndrome (1/101, 1.0%), and unspecified rheumatological disease requiring treatment (1/101, 1.0%). Some patients had more than one preexisting condition from the rheumatological or autoimmunological spectrum. The leading cardiovascular risk factors were arterial hypertension, with a proportion of 32.7%, and obesity (12.9%).

### The majority of patients had a mild course of COVID-19

To understand whether the initial severity of the course of COVID-19 might have influenced the risk of developing long-COVID, we assessed the severity of COVID-19 using the WHO clinical progression scale. The WHO progression scale ranges from 0 to 10, with a score of 1–3 representing a mild course, 4–5 a moderate disease, 6–9 a hospitalized severe disease [Intensive Care Unit (ICU) treatment], and ten representing death due to COVID-19 (12). During the acute phase of infection, 86 had a WHO clinical progression scale score ≤3, with the majority having a score of 2, indicating that patients were symptomatic and independent (78.2%; Supplementary Table 1). 14.9% of the patients had a score >3 and were thus hospitalized. 4.0% were treated in the ICU. There was no difference in the severity of COVID-19 disease depending on gender (Supplementary Figure 2A). We also found no association between age and COVID severity (*r* = 0.08, *p* = 0.43; Supplementary Figure 2B).

### Symptoms during acute infection and at the time of presentation

The most common symptoms reported during acute infection were taste disorders (67.3% of patients) and odor disturbances (65.3%). This was followed by exhaustion/fatigue (63.4%), cephalgias (51.2%), arthralgias (49.5%), and myalgias (46.5%) (Table 2). The frequency of reported symptoms changed until the time of presentation. Now, more than 90% of patients reported that they suffered from fatigue (93.1%). Moreover, a large proportion of patients reported impaired concentration (82.2%) and memory (79.2%). Sleep disturbances, only present in 28.7% of patients during the acute phase of COVID-19, increased to 53.5% at the presentation time. Impairment of smell and taste decreased to 34.7 and 27.7%, respectively.

**Table 2:** Symptoms during acute COVID-19 infection and after recovery/at time of presentation.

**Symptom**	**Acute COVID-19 infection**			**At the time of the presentation**		
	**Total *n =* 101**	**Male *n =* 27**	**Female *n =* 74**	**Total *n =* 101**	**Male *n =* 27**	**Female *n =* 74**
Exhaustion	64 (63.4%)	19 (70.4%)	45 (60.8%)	94 (93.1%)	23 (85.2%)	71 (96.0%)
Concentration impairment	na	na	na	83 (82.2%)	19 (70.4%)	64 (86.5%)
Memory impairment	na	na	na	80 (79.2%)	17 (63.0%)	63 (85.1%)
Sleep disorders	29 (28.7%)	7 (25.9%)	22 (29.7%)	54 (53.5%)	10 (37.0%)	44 (59.5%)
Odor disturbance	66 (65.3%)	11 (40.7%)	55 (74.3%)	35 (34.7%)	4 (14.8%)	31 (41.9%)
Myalgia	47 (46.5%)	8 (29.6%)	39 (52.7%)	33 (32.7%)	4 (14.8%)	29 (39.2%)
Headache	52 (51.2%)	11 (40.7%)	41 (55.4%)	31 (30.7%)	6 (22.2%)	25 (33.8%)
Arthralgia	50 (49.5%)	12 (44.4%)	38 (51.4%)	28 (27.7%)	5 (18.5%)	23 (31.1%)
Taste disorders	68 (67.3%)	14 (51.9%)	54 (73.0%)	28 (27.7%)	4 (14.8%)	24 (32.4%)
Muscle weakness	26 (25.7%)	11 (40.7%)	15 (20.3%)	23 (22.8%)	7 (25.9%)	16 (21.6%)
Paraesthesia	10 (9.9%)	3 (11.1%)	7 (9.5%)	18 (17.8%)	4 (14.8%)	14 (18.9%)
Vertigo	13 (12.9%)	2 (7.4%)	11 (14.9%)	18 (17.8%)	2 (7.4%)	16 (21.6%)
Racing heart	20 (19.8%)	4 (14.8%)	16 (21.6%)	17 (16.8%)	3 (11.1%)	14 (18.9%)
Alopecia	10 (9.9%)	1 (3.7%)	9 (12.2%)	12 (11.9%)	2 (7.4%)	10 (13.5%)
Chest pain	23 (22.8%)	5 (18.5%)	18 (24.3%)	13 (12.9%)	5 (18.5%)	8 (10.8%)
Diarrhea	25 (24.8%)	4 (14.8%)	21 (28.4%)	8 (7.9%)	1 (3.7%)	7 (9.5%)
Nausea	20 (19.8%)	4 (14.8%)	16 (21.6%)	7 (6.9%)	-	7 (9.5%)
Loss of appetite	22 (21.8%)	6 (22.2%)	16 (21.6%)	5 (5.0%)	-	5 (6.8%)
Skin rash	9 (8.9%)	3 (11.1%)	6 (8.1%)	5 (5.0%)	2 (7.4%)	3 (4.1%)
Dysphagia	9 (8.9%)	1 (3.7%)	8 (10.8%)	4 (4.0%)	-	4 (5.4%)
Vomiting	4 (4.0%)	1 (3.7%)	3 (4.1%)	1 (1.0%)	-	1 (1.4%)

Impairment of concentration and memory were not evaluated for the acute phase.

na, not available.

### Women are more severely affected by motor fatigue

**Figure 2:**
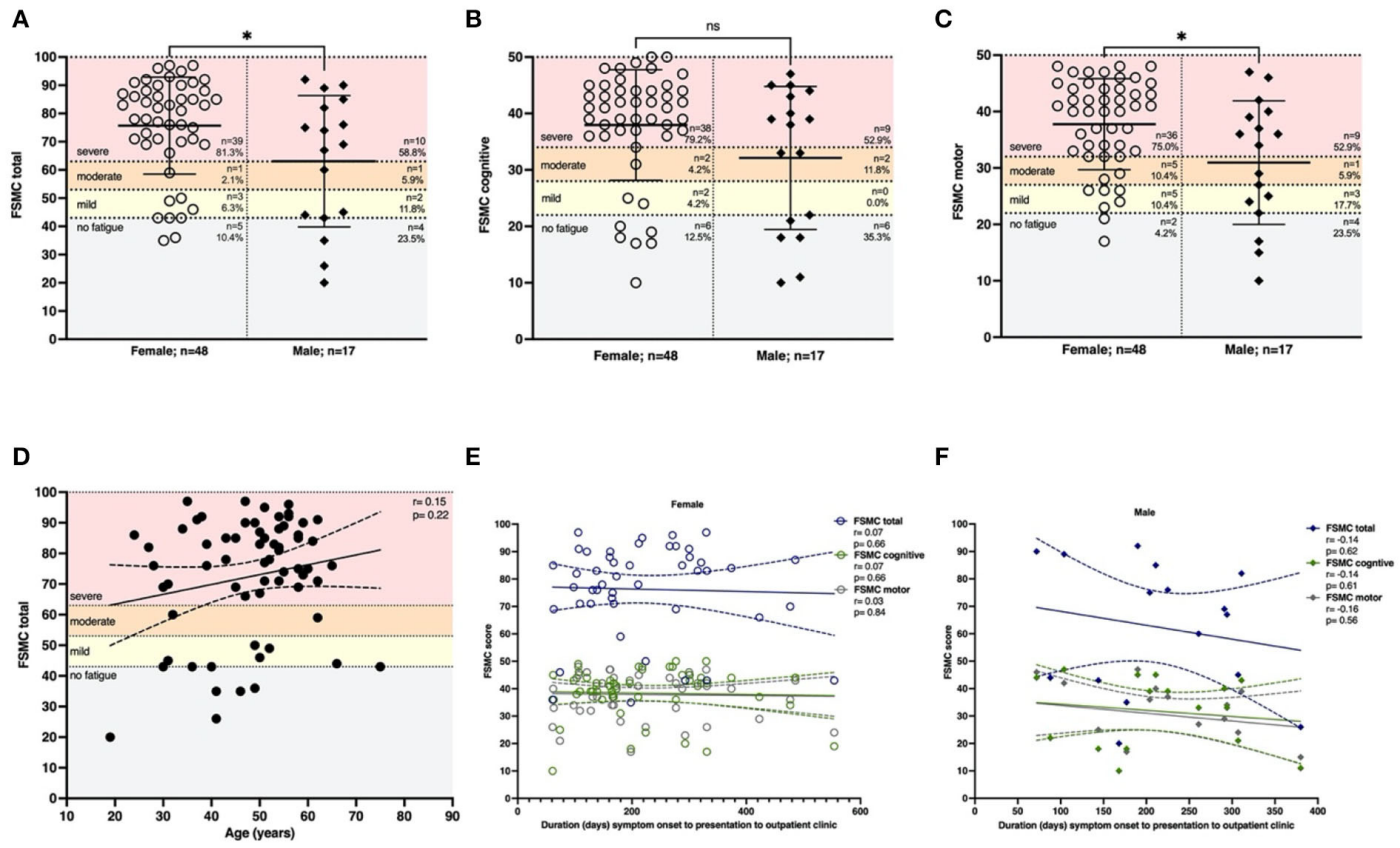
Females are affected by more severe motor fatigue. Fatigue was assessed using the FSMC score. **(A)** The majority of patients had severe fatigue (81.3% females, 58.8% males). Females were affected more severely compared to males (*p* < 0.05). This effect was driven by motor fatigue since **(B)** cognitive fatigue was not dependent on sex (*p* = 0.12). **(C)** Females were affected by more severe motor fatigue (*p* = 0.02). **(D)** Total fatigue using the FSMC was not depending on age (r = 0.15, *p* = 0.22; *n* = 65). **(E)** The latency between onset of COVID-19 and presentation did not affect the severity of total fatigue, cognitive fatigue or motor fatigue in females (total: r=0.07, *p* = 0.66; cognitive: r=0.07, *p* = 0.66; motor: *r* = –0.03, *p* = 0.84) or f) males (total: r = −0.14, *p* = 0.62; cognitive: *r* = –0.14, *p* = 0.61; motor: *r* = –0.16, *p* = 0.56). *n* = 65 total, *n* = 48 females, *n* = 17 males. Data are shown as mean ± standard deviation (SD) **(A–C)** or mean with a 95% confidence interval **(D–F)**. Data were analyzed using a two-tailed Mann–Whitney U-test **(A–C)** and nonparametric two-tailed Spearman correlation **(D–F)**. * *p* < 0.05.

Fatigue is one of the most prominent symptoms reported by patients following COVID-19. To differentiate between cognitive and motor fatigue, we took advantage of the FSMC. Incomplete questionnaires of individuals in our cohort of 101 patients were excluded. A total of 56 of 65 patients (86.2%) whose questionnaires could be evaluated had an FSMC sum score of ≥43 and, thus, at least mild fatigue symptoms (Figure 2A). Women had significantly higher total FSMC scores than men (*p* < 0.05; *n* = 48). This was reflected in a higher proportion of 81.3% of female patients with severe total fatigue compared to 58.8% of males (*n* = 17). The differentiation between cognitive and motor fatigue showed that cognitive fatigue did not depend on sex (*p* = 0.12, Figure 2B), whereas motor fatigue was significantly more pronounced in females than in males (*p* < 0.05; Figure 2C).

We then applied two logistic regression models to calculate odds ratios (OR) and the corresponding 95% confidence intervals (CI) for both fatigue sub-scores (cognitive and motor fatigue), with gender as the predictor. A value of 34 points for the subscale of cognitive fatigue and a value of 32 points for the subscale of motor fatigue was used as a cut-off to indicate severe fatigue, respectively. We adjusted for demographics (age, sex), preexisting conditions, and COVID-19 severity score according to WHO. For both outcomes (severe motor/ severe cognitive fatigue), the female sex was the only significant and independent predictor in this model. Women in our analysis had an 8-fold increased risk of suffering from severe fatigue (OR = 8.045, *p* = 0.010 for severe cognitive fatigue; OR = 7.698, *p* = 0.013 for severe motor fatigue; Table 3).

**Table 3A:** Odds ratios calculated by multivariable logistic regression model for the outcome of severe motor fatigue.

**Variable**	**OR**	**95% CI**	***P*-value**
Age	1.030	0.963–1.101	0.396
Female sex	**7.698**	**1.547–38.295**	**0.013**
Smoker	4.377	0.417–45.980	0.219
Hypertension	2.017	0.203–20.050	0.549
Diabetes	0.129	0.001–32.559	0.468
Cardiovascular disease	1.047	0.040–27.428	0.978
Cerebrovascular disease	5,013,837.486	NA	0.999
History of malignancy	966,938,006.822	NA	0.999
COPD	5,720,968.178	NA	0.999
Psychiatric or psychosomatic disease	0.828	0.106–6.464	0.857
Obesity	7.275	0.037–1,428.730	0.461
Thyroid disease	36,666,183.950	NA	0.999
Autoimmune disease	0.607	0.084–4.401	0.621
Any other disease	3.705	0.231–59.509	0.355
COVID-19 severity (WHO)	1.445	0.624–3.345	0.390
Hyperreflective lesions	471,385,404.061	NA	0.999

Significant findings are highlighted in bold.

**Table 3B:** Odds ratios calculated by multivariable logistic regression model for the outcome of severe cognitive fatigue.

**Variable**	**OR**	**95% CI**	***P*-value**
Age	1.055	0.987–1.128	0.113
Female sex	**8.045**	**1.641–39.445**	**0.010**
Smoker	2.840	0.273–29.521	0.382
Hypertension	0.712	0.095–5.353	0.742
Diabetes	0.127	0.000–113.610	0.552
Cardiovascular disease	2.015	0.104–39.008	0.643
Cerebrovascular disease	4,280,764.102	NA	0.999
History of malignancy	1.900	0.113–31.999	0.656
COPD	16,884,340.377	NA	0.999
Psychiatric or psychosomatic disease	0.717	0.092–5.579	0.751
Obesity	7.935	0.010–6,195.442	0.542
Thyroid disease	47372545.524	NA	0.999
Autoimmune disease	0.479	0.068–3.372	0.460
Any other disease	4.430	0.297–66.023	0.280
COVID-19 severity (WHO)	1.193	0.507–2.810	0.686
Hyperreflective lesions	447,271,914.738	NA	0.999

Significant findings are highlighted in bold.

To understand whether age might influence fatigue, we correlated it with age and fatigue, which showed no correlation (r = 0.15, *p* = 0.22; Figure 2D). We hypothesized that the duration of symptom onset to the time of presentation might be associated with reduced fatigue. However, the severity of fatigue was not influenced by the period between acute infection and time of presentation in women (FSMC total r = 0.07; *p* = 0.66) or men (*r* = –0.14; *p* = 0.62; Figures 2E,F).

### Depressive symptoms in males correlate with duration since symptom onset

**Figure 3:**
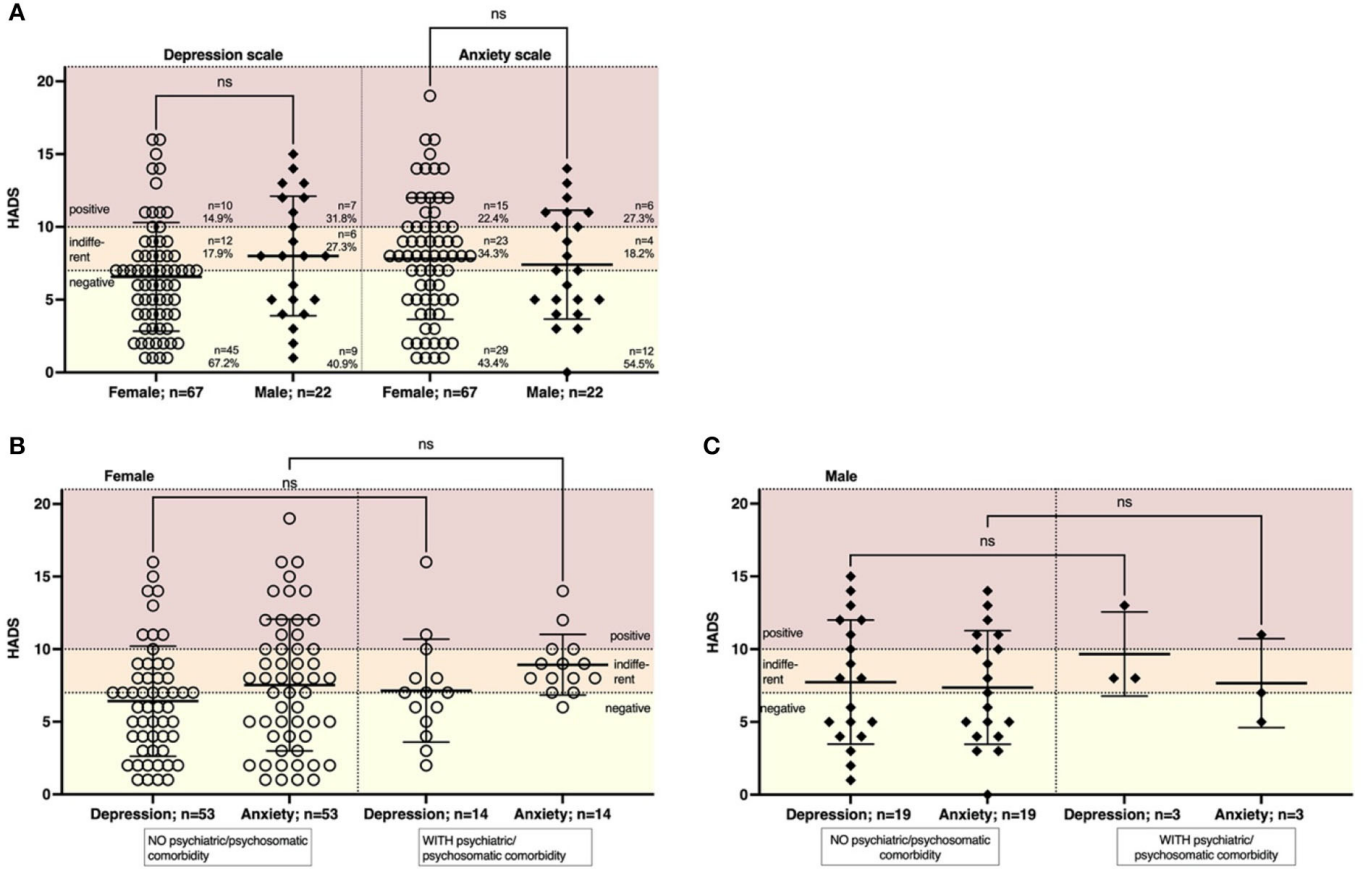
The severity of symptoms of depression or anxiety was not dependent on age or psychiatric comorbidity. We performed a HADS to assess symptoms of depression and anxiety. Scores were regarded as: 0–7 = negative, 8–10 = indifferent, >10 = positive. **(A)** The majority of patients had a negative or indifferent HADS regarding depressive symptoms (85.1% females, 68.2% males) or symptoms of anxiety (77.7% females, 72.7% males). **(B)** The severity of depressive symptoms was not affected by a previous psychiatric or psychosomatic disease in females (*n* = 14 with comorbidity, *n* = 53 women without comorbidity) or **(C)** males (*n* = 3 men with comorbidity, *n* = 19 men without comorbidity). Data are shown as mean ± standard deviation (SD). Data were analyzed using a nonparametric two-tailed Kruskal-Wallis test.

To detect symptoms associated with depression or anxiety, we performed the HADS-D (89 patients with fully completed questionnaires included). Scores ≤7 correspond to normal findings, 8–10 to suspicious findings, and scores >10 to pathological findings. 17/89 patients (19.1%, 14.9% female, 31.8% male) had pathological HADS regarding depressive symptoms. 21/89 (23.6%, 22.4% female, 27.3% male) of the patients had pathological HADS regarding anxiety symptoms (Figure 3A). There was no gender-specific difference. Likewise, there was no significant correlation with age (*r* = 0.03, *p* = 0.78 for depression, *r* = –0.09, *p* = 0.41 for anxiety). To understand whether a preexisting psychiatric or psychosomatic comorbidity might be a driving factor in the severity of depressive or anxious symptoms, we stratified patients according to comorbidities. In both females and males, the proportion of patients with preexisting psychiatric or psychosomatic comorbidity was lower compared to unaffected patients. Preexisting psychiatric or psychosomatic comorbidity did not affect depressive or anxious symptoms in females or males (Figures 3B,C). We then analyzed whether latency might be a driving factor in developing depressive or anxious symptoms. In females, both the severity of depressive symptoms (r = 0.16, *p* = 0.21) and anxious symptoms (r = 0.18, *p* = 0.14) did not depend on the latency from symptom onset to presentation (Supplementary Figures 3A,C). In males, however, we found a significant positive correlation of depressive symptoms with a duration from symptom onset (r = 0.47; *p* = 0.03; Supplementary Figure 3B), while anxious symptoms were not affected by latency (r = 0.20, *p* = 0.38).

### Impaired smell persists over time and is independent of age

**Figure 4:**
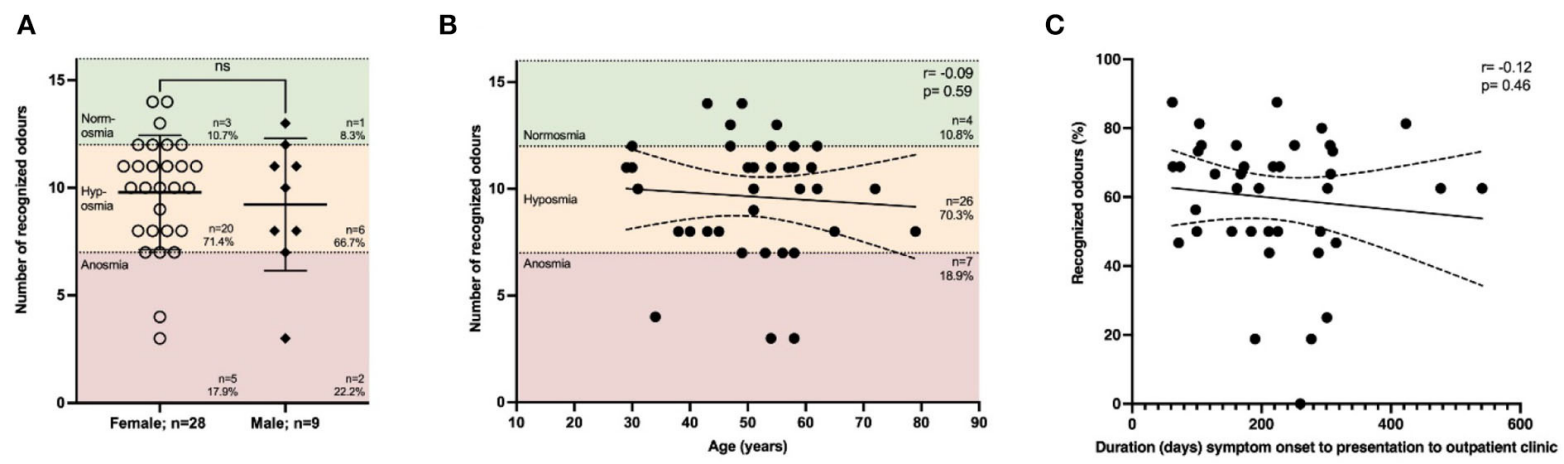
Impairment of smell is not affected by duration from symptom onset. The smell was assessed using Sniffin' Sticks^®^. The following thresholds were used: 0–7 = anosmia, 8–12 = hyposmia, >12 = normosmia. **(A)** Most patients suffered from hyposmia (71.4% females, 66.7% males), while there was no difference depending on gender. **(B)** Impaired smelling was not associated with age (*r* = –0.09, *p* = 0.59; *n* = 37 with all 16 odors tested. None of the patients recognized all 16 odors, while the maximum number of odors detected was 14 out of 16. **(C)** Duration (days) from symptom onset of acute COVID-19 infection to presentation to our clinic did not correlate (*r* = –0.12, *p* = 0.46, *n* = 40; the result of the test in percent because of varying total number of odors tested). **(A)** Data are shown as mean ± standard deviation (SD) and **(B,C)** mean with 95% confidence interval. Data were analyzed using **(A)** nonparametric two-tailed Mann-Whitney *U* test or **(B,C)** nonparametric two-tailed Spearman correlation.

Impairment of smell is one of the major symptoms of COVID-19 and was reported in 34.7% of our cohort at the time of presentation. To understand the severity of smell impairment, we performed Sniffin' Sticks on those individuals in our cohort who had anamnestic indications of an olfactory disorder (*n* = 37; female *n* = 28, male *n* = 9). The majority of patients presented with a certain degree of impaired smell, while most patients (70.3%) were categorized as being hyposmic (8–12 recognized odors) and a smaller proportion of 18.9% as being anosmic (Figure 4B). There was no gender-specific difference (Figure 4A). Age had no effect on smell (*r* = –0.09, *p* = 0.59). Moreover, we found no effect on the latency from the acute phase of COVID-19 regarding smell (*r* = –0.12, *p* = 0.46; Figure 4C). We also correlated the severity of COVID-19 according to the WHO clinical progression scale with smell, showing a modest trend that more severely affected patients might be more impaired regarding smell compared to mildly affected patients (*r* = –0.21, *p* = 0.20; Supplementary Figure 4).

## Discussion

Long-COVID or post-COVID syndrome is discussed both from a scientific point of view and increasingly in the lay media due to potential effects on many patients with multidimensional implications. Here we show that leading symptoms in the post-acute phase were fatigue, impaired concentration, and subjectively impaired memory. The most common secondary diagnosis found in our cohort was thyroid disease. Most patients with long-COVID initially had a mild disease course of COVID-19; nevertheless, most patients were affected by severe fatigue, not influenced by depressive symptoms or symptoms of anxiety. Females had an 8-fold increased risk of both cognitive and motor fatigue.

Fatigue is one of the most important symptoms found in patients with long COVID, with a prevalence of 44% in a systematic review of 39 studies (18). Other symptoms include sleep disorder (33%), dyspnea (40%), cough (22%), as well as cognitive impairment (15%), anxiety (34%), and depression (32%). Those alterations have a significant personal impact since 57% of patients reported a decreased quality of life (18). Fatigue is seen in several conditions and can also be chronic. According to the *U.S. Centers for Disease Control and Prevention* (CDC), the following mandatory and optional criteria must be met to establish a diagnosis of chronic fatigue syndrome (CFS) or myalgic encephalomyelitis (ME) (19): 1. Patients are limited by fatigue for more than six months. They cannot carry out their usual daily activities due to abnormal fatigue. 2. There is a so-called stress intolerance, also called post-exertional malaise (PEM). This means that physical, cognitive, or emotional stress leads to a decomposition of the initial symptoms. 3. patients with CFS do not have a restful sleep. Optional diagnostic criteria include difficulty concentrating, memory impairment, and orthostatic intolerance. The etiology and pathogenesis of CFS have not been conclusively clarified (20). Among others, neuroinfectious and consecutive neuroimmunological processes are discussed as potential origins of CFS. Epstein-Barr virus, human herpesvirus 6, enteroviruses, Borna disease virus, Borrelia burgdorferi, Coxiella burnetii, Candida albicans, Mycoplasma pneumonia, and retroviruses are considered possible causative agents (21). Cytokine levels might induce CFS, particularly IL-1β (22), oxidative stress, and mitochondrial dysfunction (23). Moreover, pathophysiological alterations in Long-COVID include cerebrovascular dysregulation with persistent cerebral arteriolar vasoconstriction, small fiber neuropathy and related dysautonomia, respiratory dysregulation, and chronic inflammation (24). Also discussed is autoimmune pathophysiology with the formation of autoantibodies against vasoregulatory G protein-coupled receptors in patients with CFS (25). Recently, the first report of BC007, a DNA aptamer drug with high affinity to G-protein-coupled receptors (GPCR-AAbs), showed the functional inactivation of GPCR-antibodies within 48 h after administration, leading to improvement of fatigue, taste, and retinal capillary microcirculation over four weeks in one patient (26).

One possible explanation for the development of fatigue is that patients might be more affected by affective disorders. In line with our findings, Calabria et al. found that in a group of 136 patients with cognitive complaints following COVID-19 infection, 82.3% reported fatigue, especially severe motor fatigue. Interestingly, elevated levels of apathy, anxiety, and executive dysfunction in neuropsychiatric measures and executive and attentional difficulties on cognitive tests were predictors of fatigue (27). Using the HADS, we found evidence of depressive symptoms in 19.1% of the patients, although we did not find any correlation with psychiatric or psychosomatic comorbidities, gender, or age. However, males were more likely to suffer from depressive symptoms the longer the latency was between acute infection and presentation in our clinic. One explanation might be that the persistence of long-COVID symptoms might have induced a depressive state. These data support the notion that patients with persistent long-COVID symptoms should receive diagnostic and therapeutic help to reduce the risk of developing an affective disorder. Using transcranial sonography, we found that patients with a hypoechogenic brainstem raphe structure have a 3.9-fold (95% CI 1.2–12.1) increased risk of depressive symptoms, presumably arguing for increased susceptibility to developing depressive symptoms following a stressful event such as COVID (28). Transcranial sonography could, therefore, be used to identify patients at risk of developing depressive symptoms following COVID. Moreover, data about neuropsychological deficits and fatigue are controversial. Using a comprehensive neuropsychological battery, including standardized and computerized cognitive tests and the MFIS scores (total score and cognitive fatigue score), another group failed to detect reliable neuropsychological predictors of cognitive fatigue in post-COVID patients (29).

Hyposmia and anosmia are often reported in patients with acute COVID-19 and during follow-up. While 65.3% of patients in our cohort reported impaired smell during the acute infection, the frequency dropped to 34.7% at the presentation time. In the majority of patients tested, we found some degree of hyposmia. Moreover, 70.2% (33/47) of patients who did not report having persistent hyposmia were tested to have impaired smell (hyposmia or anosmia). Interestingly, smell impairment did not correlate with age, which could have been explained by unknown preceding neurodegeneration or be a sign of reduced regeneration in older individuals. Qualitative changes in smell can persist for several months and even occur as late-onset symptoms months after full recovery (30). However, smell recovers in >90% of patients after six months (31).

There are several *limitations* to our study which need to be addressed. First, we recruited patients from our outpatient clinic. This might have induced a referral and selection bias, e.g., since patients with more severe depressive symptoms might not have been able to make an appointment, potentially underestimating the risk of COVID-associated affective disorders. On the other hand, patients with only mild long-COVID symptoms might have been missed because they did not see the need for a consultation. Another drawback is the missing control group, which should be recruited from patients without sequelae following COVID-19 and healthy, age-matched subjects.

Moreover, we assessed patients only at one time point; hence, follow-up studies are needed to understand the dynamics of alterations over time. Age-related neurodegeneration, which might have influenced, for example, hyposmia, was not taken into account. Moreover, we did not evaluate cognitive function systematically in this cohort, which should be addressed in further studies. The strength of the data presented here is the sufficiently large number of patients investigated and the conclusive acquisition of data, including socioeconomic and clinical data.

Whether long-COVID is a distinct disease entity with unclear pathophysiology or a spectrum of prolonged viral infection remains unclear. The scientific and media attention induced by COVID-19 almost pushes us to consider symptoms in the post-acute phase as a separate disease entity. However, a critical appraisal of the literature also implies that at least part of the symptoms in the post-acute phase of SARS-CoV-2 infection, namely fatigue, might be a spectrum of CFS with SARS-CoV-2 virus as another virogenic etiology. Persisting alteration of smell, however, seem to be rather specific to SARS-CoV-2 infection.

*In summary*, we provide a holistic picture of patients with long-COVID presenting in a specialized neurology university hospital setting and show that patients with long-COVID syndrome and mild disease are affected by severe fatigue, with an 8-fold increased risk in women. Further studies, including larger sample sizes, control groups, and longitudinal designs, are needed to better understand the dynamics of long-COVID over time.

## Data availability statement

The raw data supporting the conclusions of this article will be made available by the authors, without undue reservation.

## Ethics statement

The study was conducted in accordance with the Helsinki Declaration of 1975 and was approved by the Local Ethics Committee of Ruhr-University Bochum (20-6827 and 21-7423). The patients/participants provided their written informed consent to participate in this study.

## Author contributions

HS, JCJ, NT, DR, TP, NS, and IA: revising the manuscript and acquisition of data, analysis, and interpretation of data. RG: revising the manuscript, analysis, and interpretation of data. SF: writing the manuscript, acquisition of data, analysis, and interpretation of data, study concept and design, and study supervision. All authors read and approved the final version of the manuscript.

## Conflict of interest

The authors declare that the research was conducted in the absence of any commercial or financial relationships that could be construed as a potential conflict of interest.

## Publisher's note

All claims expressed in this article are solely those of the authors and do not necessarily represent those of their affiliated organizations, or those of the publisher, the editors and the reviewers. Any product that may be evaluated in this article, or claim that may be made by its manufacturer, is not guaranteed or endorsed by the publisher.

## Abbreviations

ACE-2-Receptor: Angiotensin-Converting Enzyme 2 Receptor
ART: Automatic Real-Time
CDC: U.S. Centers for Disease Control and Prevention
CFS: Chronic Fatigue Syndrome
CI: Confidence Interval
COPD: Chronic Obstructive Pulmonary Disease
COVID-19: Coronavirus Disease 2019
DNA: Deoxyribonucleic Acid
FSMC: Fatigue Scale for Motor Function and Cognition
GPCR-A: G Protein-Coupled Receptor A
HADS: Hospital Anxiety and Depression Scale
ICU: Intensive Care Unit
IFN-1β: Interferon 1 β
iNOS: Inducible Nitric Oxide Synthase
ME: Myalgic Encephalomyelitis
OR: Odds ratio
PEM: Post-Exertional Malaise
pRNFL: peripapillary Retinal Nerve Fiber Layer
SARS-CoV-2: Severe Acute Respiratory Syndrome Coronavirus 2
SD-OCT: Spectral-Domain Optical Coherence Tomography
SD: Standard Deviation
TNF-α: Tumor Necrosis Factor α
WHO: World Health Organization.
