# Supplementary material for: Cross-sectional analysis of clinical aspects in patients with long-COVID and post-COVID syndrome
Source: Front Neurol. 2022 Oct 14;13:979152. doi: 10.3389/fneur.2022.979152 (PMC9614029; doi:10.3389/fneur.2022.979152)
Supplement: Supplementary file 1 [file Data_Sheet_1.docx]

**SUPPLEMENT**

**Supplementary Table 1: Data on course of disease and therapeutic measures during acute COVID-19 infection.**

|  | Total | Male | Female |
| --- | --- | --- | --- |
| Hospitalization | 14/95 (14.7%) | 5/25 (20.0%) | 9/70 (12.9%) |
| Intensive care | 4/93 (4.3%) | 1/24 (4.2%) | 3/69 (4.3%) |
| Oxygen therapy  Non-invasive or mask ventilation | 5/93 (5.4%)  2/93 (2.2%) | 2/24 (8.3%)  - | 3/69 (4.3%)  2/60 (2.9%) |
| Antibiotics | 11/90 (12.2%) | 4/24 (16.7%) | 7/66 (10.6%) |
| Antivirals | 3/87 (2,94%) | 1/23 (3,70%) | 2/64 (2,67%) |
| Corticosteroids | 4/88 (4.5%) | 1/24 (4.2%) | 3/64 (4.7%) |
| WHO clinical progression scale  Ambulatory mild disease  Score 1  Score 2  Score 3  Hospitalized moderate disease  Score 4  Score 5  Hospitalized severe disease  Score 6  Score 7 | 4/101 (4.0%)  79/101 (78.2%)  3/101 (3.0%)  9/101 (8.9%)  6/101 (5.9%)  -  - | 3/27 (11.1%)  17/27 (63.0%)  1/27 (3.7%)  3/27 (11.1%)  3/27 (11.1%)  -  - | 1/74 (1.4%)  62/74 (83.8%)  2/74 (2.7%)  6/74 (8.1%)  3/74 (4.1%)  -  - |

**SUPPLEMENTARY FIGURES**

**
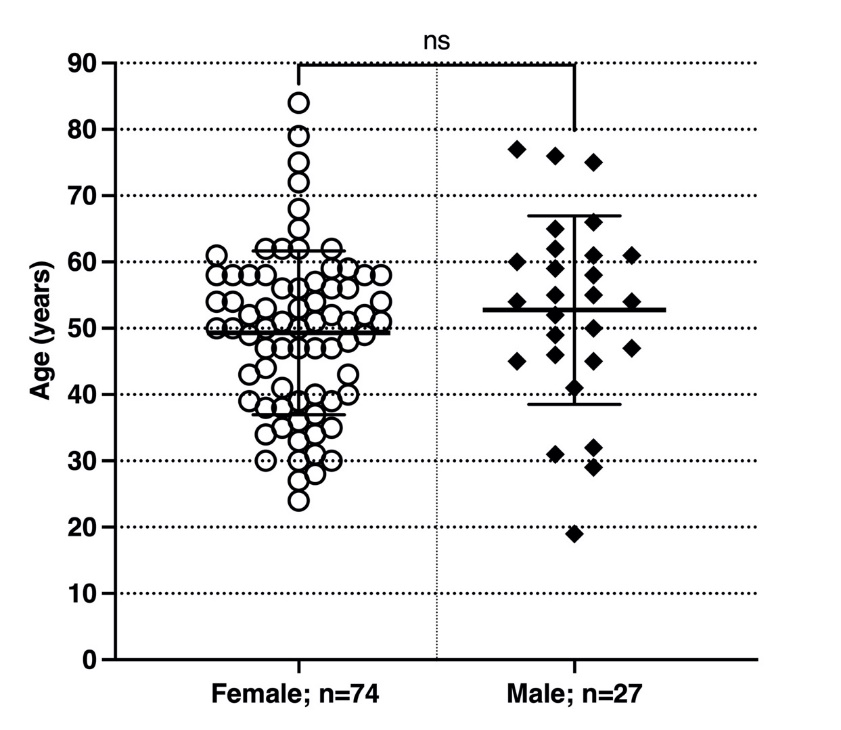
**

**Supplementary Figure 1: Distribution of age depending on sex.** The mean age was 49.3 years (range 24-84 years) in females and 52.7 (range 19-77 years) in males. Females n=74, males n=27. Data are shown as mean ± standard deviation (SD). Data were analyzed using non-parametric two-tailed unpaired t test.


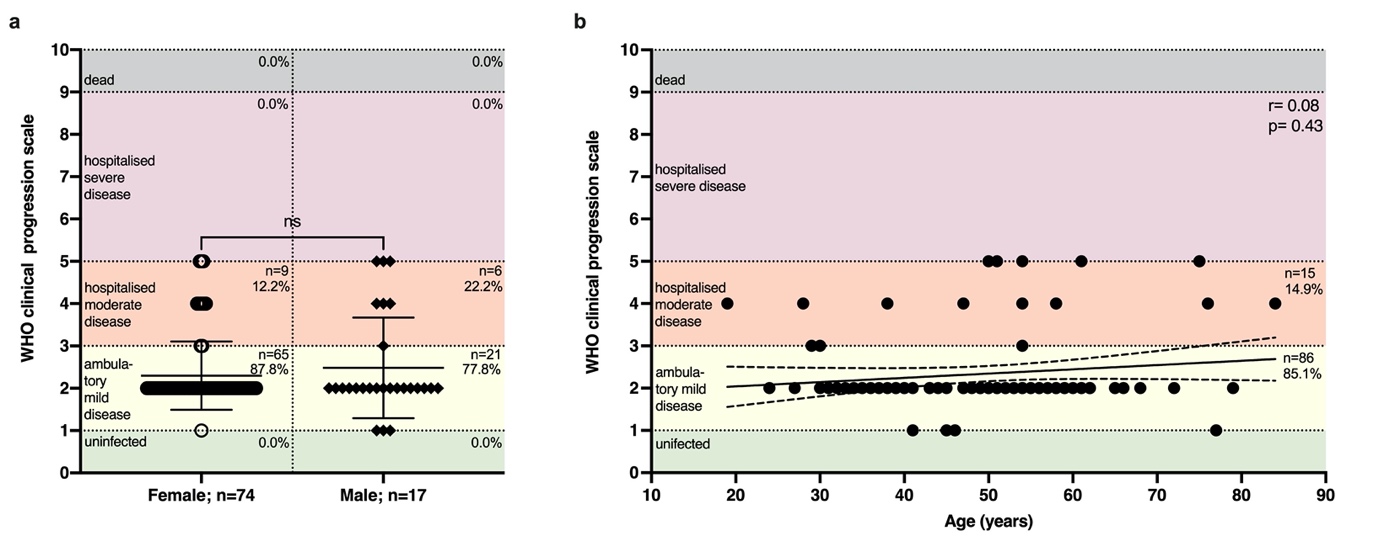


**Supplementary Figure 2: Assessment of COVID-19 severity according to WHO clinical progression scale.** a) There was no difference regarding severity of COVID-19 between females and males. Most patients had a mild disease course (87.8% of all women, 77.8% of all men). b) Age and disease severity did not correlate (r=0.08, p=0.43). a) Data are shown as mean ± standard deviation (SD) and b) mean with 95% confidence interval. Data were analyzed using a) non-parametric two-tailed Mann-Whitney U test or b) non-parametric two-tailed Spearman correlation.

**
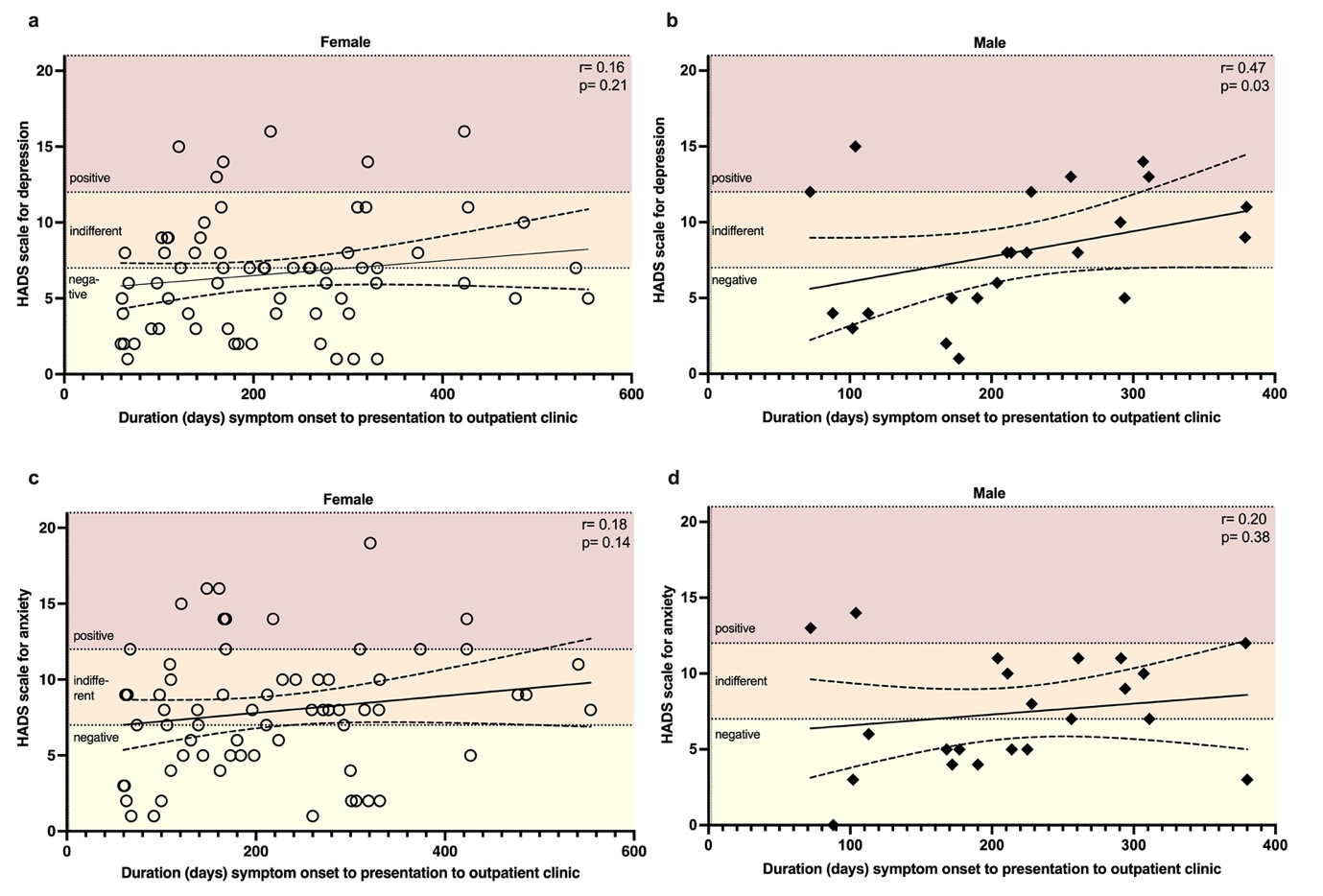
**

**Supplementary figure 3: Males had more severe depressive symptoms with increased duration from symptom onset.** We correlated duration (days) from symptom onset to presentation to the outpatient clinic and the severity of depressive and anxious symptoms. Scores were regarded as: 0-7=negative, 8-10=indifferent, >10=positive. Duration from symptom onset did not correlate with both a) depressive symptoms (r=0.16, p=0.21) and c) anxious symptoms (r=0.18, p=0.14) in females. In males, however, there was a positive correlation of duration of symptom onset to presentation with b) depressive symptoms (r=0.47; p=0.03) while d) anxious symptoms were not affected (r=0.20, p=0.38). Data are shown as mean with 95% confidence interval. Data were analyzed using non-parametric two-tailed Spearman correlation.


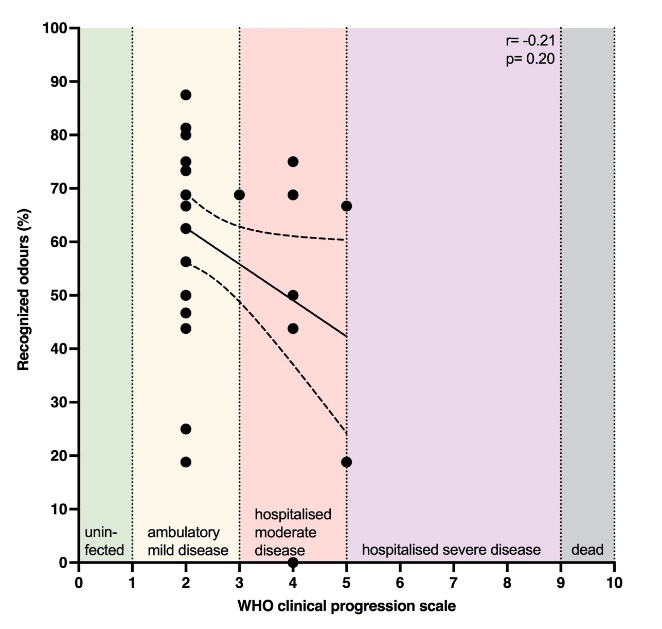


**Supplementary Figure 4: Severity of COVID-19 disease did not correlate with impaired smell.** There was no correlation between severity of acute COVID-19 disease (measured by assessing the WHO clinical progression scale) and percentage of recognized odors assessed using Sniffin Sticks® (r=-0.21, p=0.20). Data are shown as mean with 95% confidence interval. Data were analyzed using non-parametric two-tailed Spearman correlation.
